# KidsTUMove—A Holistic Program for Children with Chronic Diseases, Increasing Physical Activity and Mental Health

**DOI:** 10.3390/jcm13133791

**Published:** 2024-06-28

**Authors:** Nicola Stöcker, Dominik Gaser, Renate Oberhoffer-Fritz, Christina Sitzberger

**Affiliations:** 1 TUM School of Medicine and Health, Department Health and Sport Sciences, Applied Sciences, Technical University of Munich, 80809 Munich, Germany; 2 TUM School of Medicine and Health, Department Health and Sport Sciences, Preventive Pediatrics, Technical University of Munich, 80992 Munich, Germany; dominik.gaser@tum.de (D.G.); renate.oberhoffer@tum.de (R.O.-F.)

**Keywords:** congenital heart diseases, cancer, survivor, illness, adolescents, pediatrics, sports, quality of life, self-esteem, empowerment, KidsTUMove

## Abstract

The prevalence of chronic diseases in children and adolescents has risen alarmingly worldwide. Diseases such as asthma, diabetes, obesity, mental disorders, and congenital heart defects are increasingly affecting the lives of children and pose significant challenges for the healthcare system. Physical activity plays a crucial role in preventing and treating these diseases. Numerous studies have shown that regular exercise improves physical performance, increases well-being, and leads to better health in the long term. Specially tailored sports programs that meet the individual needs and abilities of the children and adolescents affected are particularly important. The KidsTUMove project addresses this by developing tailored exercise programs for children with chronic diseases’ specific needs, medical conditions, and physical abilities. Therefore, it closes the gap in care provision and can thus sustainably improve the health prospects of these children and adolescents. KidsTUMove is positioned to make a significant impact on the lives of affected children across Europe. Promotion of such programs should therefore be an integral part of future health strategies.

## 1. Introduction

Over the past 30 years, the prevalence of chronic diseases in children and adolescents has increased [1]. Twenty-five percent of children worldwide suffer from a chronic disease [2]. The prevalence among US children shows an increase in the disease rate from 12% in 1994 to 26.6% in 2006 [3]. Similarly, in Germany, approximately one in six children aged 0 to 17 years suffers from a chronic disease. [4]. An enhanced examination of the bio-psycho-social consequences of physical inactivity within this subgroup becomes essential. Global challenges, including the compounding effects of the COVID-19 pandemic and instances of conflict, have accentuated the vulnerabilities of children with chronic diseases [5,6,7]. Physical activity and sports play a significant role in childhood development [8,9,10]. However, contemporary lifestyles have given rise to a concerning trend of physical inactivity among children and adolescents [11,12,13]. The majority do not meet current physical activity guidelines [14]. This lack of physical activity contributes to developing conditions such as overweight and obesity, ultimately leading to secondary diseases like diabetes mellitus and cardiovascular disorders [15,16,17,18]. In contrast, children and adolescents who engage in higher levels of physical activity exhibit better physical and mental health and improved psycho-social well-being compared to their inactive peers [19]. The current global physical activity recommendations by health authorities, such as the World Health Organization (WHO), emphasize the importance of regular physical activity for optimal health outcomes. However, an examination of children’s adherence to these guidelines reveals a substantial gap, particularly among those with chronic diseases [20,21] Although international physical activity guidelines provide recommendations for healthy children [22], there is a lack of disease-specific guidelines for pediatric patients with chronic diseases. Despite this gap, regularly practiced exercises are feasible and safe for most children with chronic diseases [23,24] and positively influence risk factors for sequelae and secondary diseases [5]. One reason for physical inactivity among children with chronic diseases is overprotection by parents, caregivers, and teachers, who aim to avoid a perceived overload, often leading to exemptions from physical education [25,26]. For most children, physical education serves as the first step back into sporting activities after therapy or surgery. However, there is a lack of supportive sports or club sports programs to reintegrate children into sports after long periods of illness [27,28]. To achieve the WHO guidelines, more implementation options must be available in most countries, including Germany [29,30].

Based on responses from the families involved, it is frequently observed that children and adolescents receive minimal sporting support following discharge from inpatient rehabilitation, leaving them to self-manage their physical activity endeavors [31]. In most cases, parents’ commitment plays a significant role in ensuring that the children can take advantage of further offers. However, socially disadvantaged or low-income families face challenges and disadvantages, including barriers to engaging in regular sports participation [32,33].

To the best of our knowledge, sports programs addressing the unique needs of children and adolescents with chronic diseases are only rarely available in Germany. There is a lack of national and European standards that regulate the content of targeted sports programs and funding opportunities [34,35]. KidsTUMove serves as a best practice for holistic sports intervention programs in Germany. This report outlines an innovative strategy to promote physical activity to enhance the overall well-being of children and adolescents with chronic diseases [36]. 

## 2. Aims and Scopes

An essential aspect of addressing the challenges faced by children with chronic diseases is the development of tailored exercise programs specific to their individual needs, medical conditions, and physical abilities. These programs aim to provide targeted interventions that optimize health outcomes and improve the quality of life for these children.

**Figure 1:**
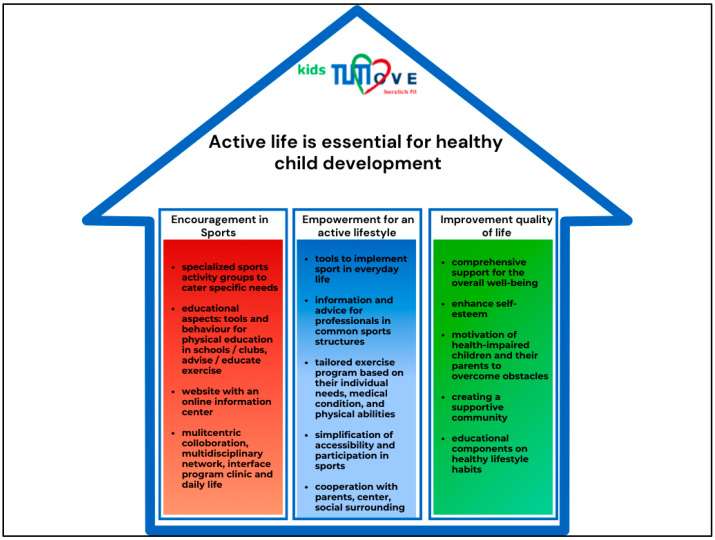
Illustration of the aims and scopes of KidsTUMove. Own presentation.

The primary goal of the healthcare project KidsTUMove, with its modular program, is to foster physical activity among children, providing them with an impulse in a safe and supportive environment where they can participate in age-appropriate activities and interact socially with peers facing similar challenges [37]. Through engagement in these activities, children can cultivate confidence, improve their self-esteem, and enhance their overall well-being [38]. The project encompasses a spectrum of physical activities offered year-round and provides comprehensive support for the holistic well-being of the children. This holistic approach may entail educational components addressing healthy lifestyle habits, access to counseling services, and provision of resources aimed at assisting children and their families in effectively managing their conditions. The modular program offers a diverse array of activities meticulously tailored to accommodate each child’s specific needs and abilities. Its overarching aim is to offer support and guidance to these children and their families, aiding them in navigating the challenges associated with their conditions. By fostering a supportive community, the modular program facilitates social connections among participants, facilitating the sharing of experiences and mutual encouragement. In order to establish the needs of children with chronic diseases, an analysis of their living situation serves as the foundation for understanding the unique challenges and circumstances (see Figure 1). Scopes like physical activity and education, scientific evidence, promotion of self-esteem, and involvement of parents are adapted in the holistic healthcare project KidsTUMove to meet the specific needs.

### 2.1. Encouragement in Children with Chronic Diseases through Specialized Sports Activities and Personal Support

**Figure 2:**
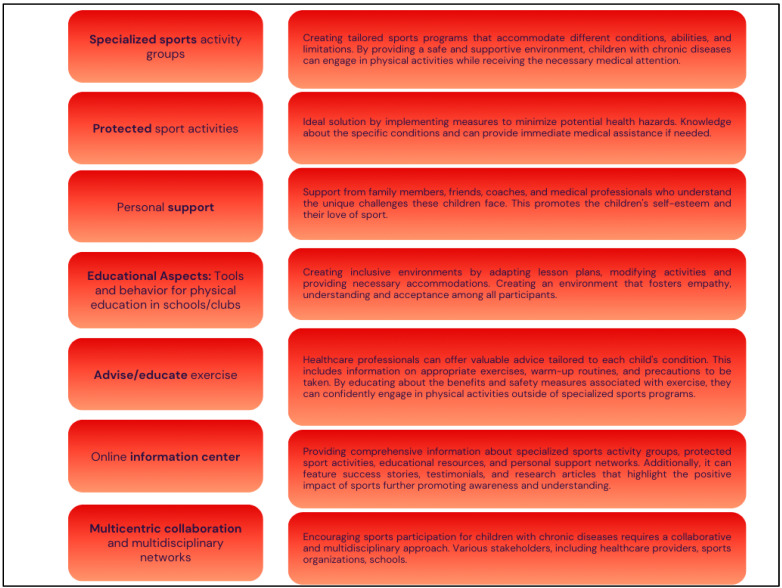
Implementation of the own encouragement in children with chronic diseases. Own illustration.

Encouraging children with chronic diseases through specialized sports activities and personalized support can significantly enhance their physical and emotional well-being, fostering a sense of accomplishment and resilience. Figure 2 provides insights into the different aspects of achieving positive impacts of these interventions. Engaging in sports and physical activities benefits a child’s physical health and is crucial for their emotional well-being and overall development [39]. Thus, for example, motor performance decreases in children with cancer during the progression of treatment and affects physical activity with side effects and long-term consequences [40]. Similarly, a Brazilian study showed that the predictor of obesity influences motor skills and physical performance in children and adolescents [41] and physical performance [42]. For children living with chronic diseases, participating in sports may seem challenging due to the specific needs and limitations associated with their conditions. However, with positive encouragement, specific sports activities, and personal support, these children can experience the joys and benefits of sports just like their peers. This part explores various aspects of encouragement in sports for children with chronic diseases and highlights the importance of creating an inclusive environment for their participation.

### 2.2. Empowering Children Leading an Active Life

**Figure 3:**
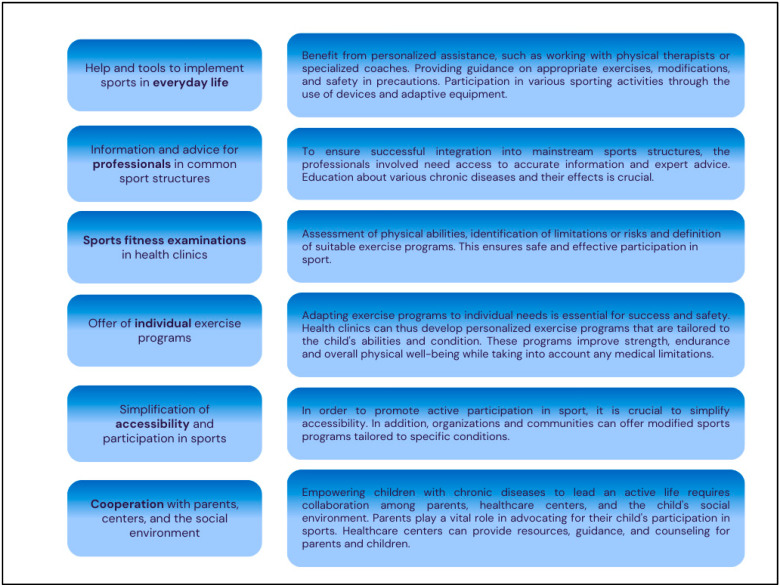
Implementation of empowering children to lead an active life. Own illustration.

Children with chronic diseases often face difficulties in leading active and fulfilling lives. However, they can fully participate in regular physical activities and sports with the appropriate support, resources, and information. The healthcare project KidsTUMove, with its modular program, empowers children with chronic diseases to maintain active lifestyles by offering assistance, tools, and guidance to incorporate sports into their daily routines. It also emphasizes the importance of sports fitness examinations in health clinics [43], offers individual exercise programs, simplifies accessibility, and fosters cooperation with parents, centers, and the social environment. By integrating sports into daily routines, offering personalized exercise plans, conducting fitness assessments, and fostering collaboration with parents and the community, the program aims to help children with chronic diseases enjoy regular physical activity’s physical and emotional benefits. This approach fosters a community that embraces inclusivity and empowers children with chronic diseases to lead active and fulfilling lives. Figure 3 shows the implementation.

### 2.3. Enhancing Quality of Life for Children with Chronic Diseases: Comprehensive—Support for Overall Well-Being

**Figure 4:**
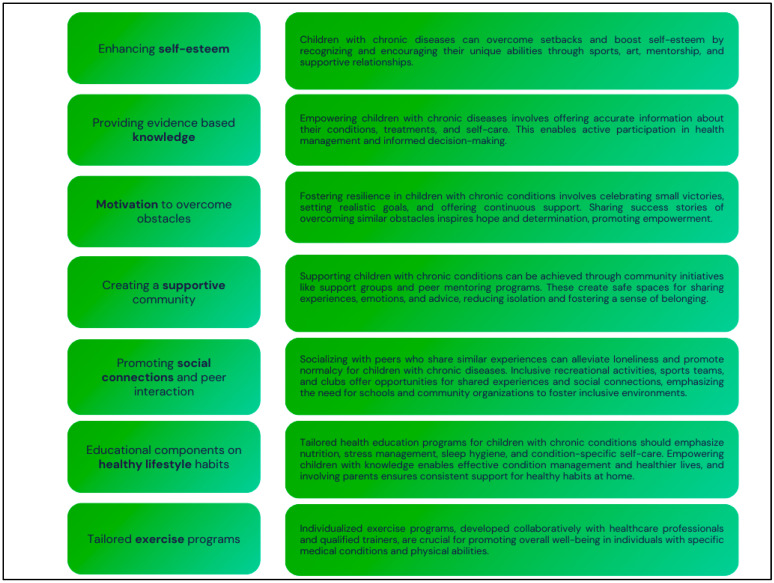
Implementation of enhancing quality of life. Own illustration.

As already stated in the Introduction, children with chronic diseases face unique challenges that can significantly impact their quality of life [44,45,46,47,48]. However, comprehensive support systems, tailored exercise programs, and a nurturing community can improve their overall well-being. Additionally, educational components on healthy lifestyle habits and personalized exercise programs based on individual needs and medical conditions are crucial in improving their quality of life. Figure 4 shows the implementation.

## 3. Materials and Methods

### 3.1. Program Design and Participant Recruitment

The healthcare project KidsTUMove includes children with chronic diseases. All subjects, including children and their legal guardians, have given their informed consent for inclusion before participating in the program. KidsTUMove as a healthcare project was approved by the Ethics Committee of Technical University of Munich (373/15s). All participants are screened according to a standardized protocol [36]. 

Inclusion Criteria:

- All participants aged 4–18 with a chronic disease;
- Medical certification of fitness for physical activity;
- Parental/guardian consent for participation.

Exclusion Criteria:

- Deterioration in general health;
- Injuries and acute illnesses (Infections, diseases, open wounds).

The modular program aims to engage and educate young individuals about the importance of an active lifestyle through diverse activities and approaches. Effective recruitment is essential to raise awareness of the KidsTUMove program among children, adolescents, and their families. This can be achieved through direct engagement in clinics via doctors’ recommendations, health insurance companies during pre- or post-rehabilitation, and word-of-mouth advertising. An extensive network, including pediatricians, school psychologists, social workers, and parent initiatives in Bavaria, is utilized to inform affected children about KidsTUMove. 

Once contact is established, children and adolescents are enrolled in the KidsTUMove sports programs. A distinctive feature of the programs is the involvement of a multidisciplinary team, including medical, psychological, and sports experts, as well as parents and the affected child, to discuss the requirements, goals, and aspirations for maintaining a healthy, active lifestyle. Detailed medical history interviews and consultations with treating physicians are conducted, and children can continue participating in KidsTUMove programs until they reach adulthood.

The multidisciplinary team’s specialized knowledge enables KidsTUMove to provide optimal physical activity for children and adolescents with chronic diseases. Operating within the ecosystem of the Technical University of Munich, Germany, KidsTUMove leverages its resources and expertise in sports science and health education. The program includes a consortium of experts that contribute to its success:

- Sports science professionals: Sports scientists lead the multidisciplinary team and investigate the effects of physical activity on children with chronic diseases. They develop the program’s sports education concept and organize, evaluate, and carry out the sports activities.
- Health science professionals: KidsTUMove recognizes the importance of collaboration with health science professionals within the healthcare system. They contribute their expertise in pediatric health, nutrition, and psychology, providing valuable insights and ensuring the program aligns with evidence-based practices. This collaboration ensures that KidsTUMove remains at the forefront of health education for children.
- Pediatricians: Pediatricians and sports physicians assess the physical fitness of the participants and provide medical care during the sporting activities.
- University students: KidsTUMove benefits from a large group of university students who have the opportunity to learn about the program’s concepts and principles. Through their involvement, they gain valuable experience and knowledge in promoting physical activity among children. These students act as ambassadors and can further disseminate the program’s concepts to the broader community.
- Exercise professionals and coaches: As KidsTUMove expands its reach, it aims to establish partnerships with schools and educational institutions to create a sustainable framework. The framework involves training and supporting coaches in delivering the program’s activities. Coaches are trained in child development, physical education, and health promotion. They serve as role models and mentors for the children, guiding them toward a more active lifestyle.

### 3.2. Program Evaluation and Assessments 

The program evaluation section delineates the project’s holistic approach’s foundational components and practical implementations. This includes motor performance tests [49], questionnaires assessing health-related quality of life and well-being [50,51], and monitoring of physical activity using accelerometers [52]. It is imperative that all program components, including KidsTUMove (KTM), undergo regular and professional evaluation. Evaluations are conducted routinely during camps (for parents and participants), before and after each camp session. Additionally, annual discussions are held with all families, and program evaluation is carried out by participating students. Internal team evaluations are also conducted to ensure ongoing refinement and optimization of the program. 

## 4. Results

No results of scientific data analyses are presented in this project report. Instead, this section focuses on the presentation of the developed programs, contents, and methodological concepts. Results on the positive effects of the holistic healthcare project KidsTUMove on motor performance and health-related quality of life among program participants will be published soon.

### 4.1. Program Overview

KidsTUMove was founded in 2007 by experts of the Chair of Preventive Pediatrics at the TUM School of Medicine and Health, Department of Health and Sport Sciences at the Technical University of Munich, Germany [36]. Since 2010, 2750 participants have taken part in the KidsTUMove programs. Initially, KidsTUMove focused on promoting physical activity and improving the quality of life for children with congenital heart diseases (CHD). Following the program’s implementation in cooperation with the German Heart Center Munich, the offerings have expanded since 2011 to include childhood cancer survivors, children with obesity, asthma, diabetes, and rheumatic diseases, and socially conspicuous children.

**Figure 5:**
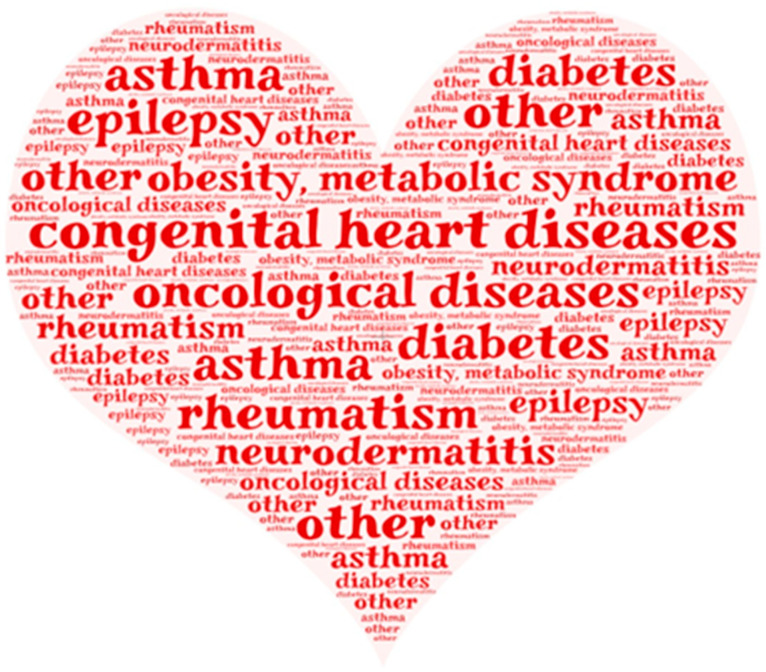
Word cloud depicting various chronic diseases among KidsTUMove participants. Own illustration.

Figure 5 shows the diversity of chronic diseases the participants suffer from in a word cloud (a visual representation of chronic diseases where more frequently occurring diseases are highlighted with greater prominence).

**Figure 6:**
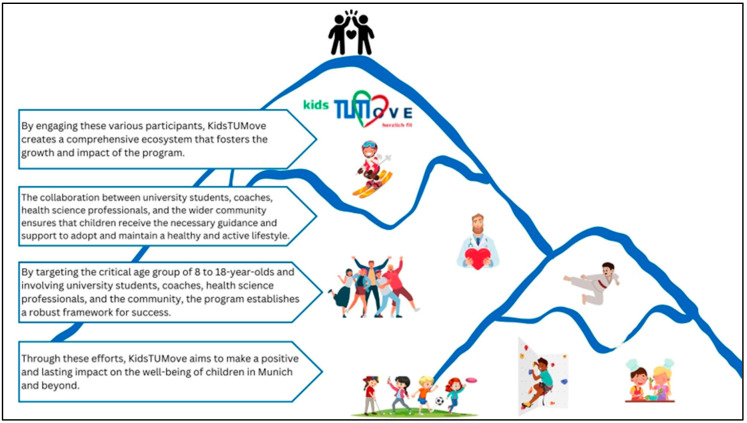
Holistic healthcare project KidsTUMove. Own illustration.

The program evaluation has demonstrated that children and adolescents experienced an improved quality of life after participating in the KidsTUMove program, with these benefits persisting over the long term for several months [53,54,55,56]. The provision of KidsTUMove sports activities for children with chronic diseases is safely achievable through professional supervision and deliberate, individualized load control, enabling children to engage in all sporting activities with the knowledge about individual adaptation (material, game rules). Opening sports programs to all children, regardless of diagnosis, has proven effective. It should be particularly emphasized that children exhibit mutual respect and sensitivity toward diverse medical histories. Continuous consultation with pediatricians is essential for assessing stress thresholds, identifying unsuitable sports, and monitoring medication regularly. Positive feedback from parents and children is highlighted, including testimonials such as “bright children’s eyes” or released parents who have learned that conveying anything outside conventional norms can be challenging. In addition, a KidsTUMove Friends Club of former participants (>18 years of age) is creating a new network with the unique flair embedded and experienced within KidsTUMove’s programs. Figure 6 shows long-term goals and internal mechanisms for achieving the objectives. 

The following presents the individual components (= modular program) of the holistic healthcare project KidsTUMove.

### 4.2. Move It—Integrative Sports Group

The Move it—integrative sports group distinguishes itself from standard sports groups by offering a unique and individualized approach to physical activity for children. Unlike larger sports programs, Move it emphasizes small groups led by qualified trainers who customize programs to each child’s needs, considering factors such as medical conditions and physical abilities. The group’s distinctive feature is its diverse range of sports activities, which exposes children to various physical disciplines. The program values incorporating rituals, fostering a sense of routine and engagement without unnecessary pressure. The focus is on creating a fun atmosphere where children can develop physical skills, socialize with peers, and cultivate a positive attitude toward physical activity. By prioritizing individualized attention, a variety of sports, and a light-hearted approach, Move it ensures children a well-rounded and enriching experience beyond the conventional sports group model. 

### 4.3. Summer and Winter Camps

KidsTUMove summer and winter camps redefine the camp experience by offering a combination of high-quality care, a secure environment, and customized programs for varying age groups. With a low child-to-staff ratio, KidsTUMove ensures personalized and attentive care. These camps prioritize round-the-clock medical services, providing parents with peace of mind and fostering close collaboration between camp staff and parents. The camp facilities are designed to create a comfortable atmosphere with a strong connection to nature. The respectful and nurturing environment promotes a sense of belonging, serving as the backdrop for diverse programs led by qualified trainers. These trainers design individualized programs for each child, taking into account their specific needs and abilities. The camps feature a variety of sports, inclusive rituals, and activities focused on enjoyment rather than performance pressure. The emphasis on fun is central, ensuring that children develop physical skills and cultivate positive attitudes toward sports. 

### 4.4. Weekend Events

KidsTUMove weekend events offer a break from routine while promoting personal growth and well-being. These events encompass a diverse array of activities spanning various themes, such as self-defense and being a “superhero today”. The primary focus is on establishing a welcoming and supportive atmosphere, enabling children to engage in various sports and recreational pursuits at their own tempo. The low-stress environment ensures that enjoyment and fun take precedence, affording children a weekend replete with enriching encounters, favorable social engagements, and the chance to cultivate a lasting affinity for physical activity. 

### 4.5. Climbing Group 

The KidsTUMove climbing group offers children an enriching experience characterized by expert guidance, small-group dynamics, and a supportive environment. Led by qualified trainers, children receive personalized instruction to ensure a safe and effective introduction to climbing. The small-group format fosters a secure and protected felt atmosphere where each child receives individualized attention, enabling trainers to tailor their approach to match each child’s abilities and objectives. This intimate setting facilitates meaningful interactions, allowing trainers to engage with children and build connections beyond the climbing wall. Moreover, the KidsTUMove climbing group extends beyond physical activity; it integrates the goals and skills learned during sessions into children’s daily lives. This holistic approach ensures that the benefits of climbing extend beyond training sessions, empowering children to apply newfound confidence and problem-solving abilities to various aspects of their routines. In summary, the climbing group provides a comprehensive and supportive platform for children to explore, learn, and grow within the exciting realm of climbing. 

### 4.6. KidsTUMove Goes Online!

**Figure 7:**
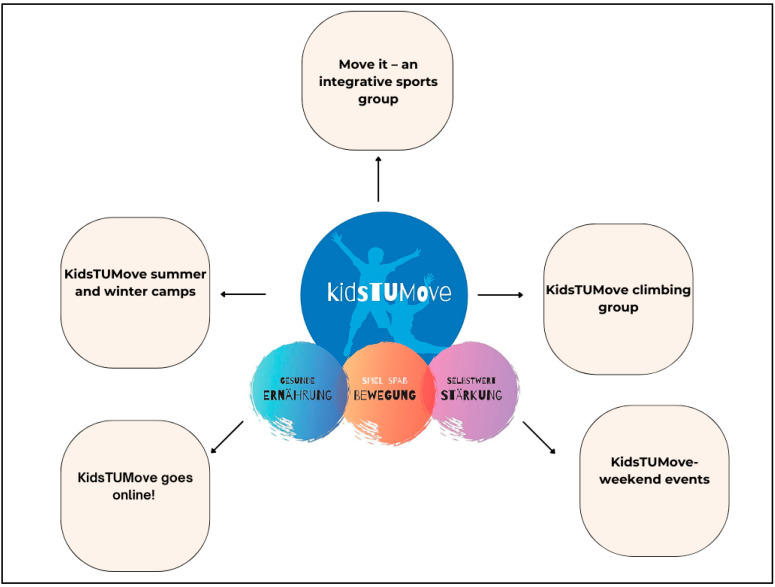
Modular program offer of KidsTUMove. Own presentation.

This online initiative functions as a supportive and motivational platform, promoting physical activity while disseminating health-related information. Through its transition to an online format, KidsTUMove goes online! aims to remotely accompany and motivate children, addressing the constraints imposed by the COVID-19 pandemic and reaching individuals facing geographical or logistical barriers to in-person participation. This adaptation not only facilitates physical activity but also serves as a means of delivering health education and maintaining personal connections with participants. Such adaptability enhances the program’s capacity to positively influence the well-being of children, even amidst challenging circumstances. Figure 7 shows all offers of KidsTUMove.

### 4.7. Tailoring KidsTUMove Activities for Pediatric Chronic Diseases

**Figure 8:**
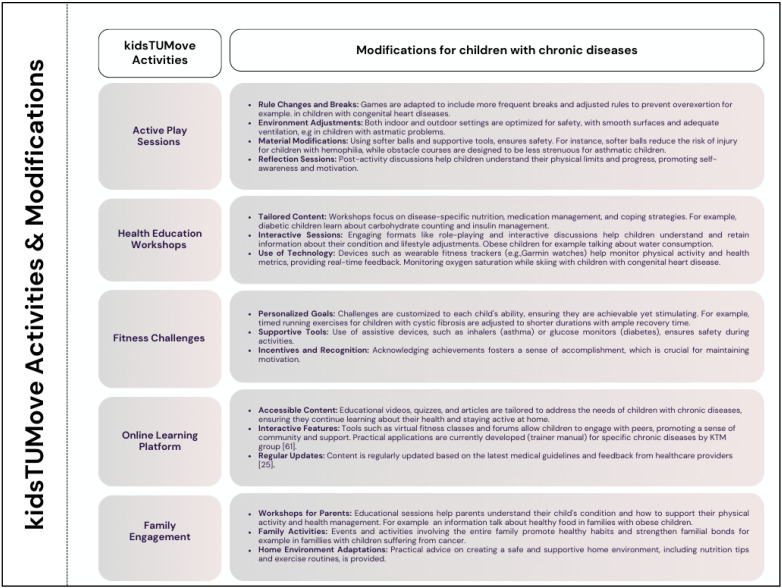
KidsTUMove activities and modification. Own presentation.

As previously stated in this report, the KidsTUMove program integrates numerous activities designed to promote physical health and well-being in children. These activities are carefully customized to accommodate children with chronic diseases, emphasizing inclusivity and safety while optimizing health outcomes. To equip trainers with appropriate knowledge, the KTM group [57] will publish a manual focusing on specific chronic diseases and physical activity, drawing from established guidelines [24,58,59]. The adjustments are informed by practical applications of exercise as medicine, as outlined in the literature [24]. Figure 8 illustrates examples of adaptations for each activity. 

### 4.8. Funding and Resource Allocation

To ensure the sustainability and scalability of KidsTUMove, strategic funding and resource allocation have been implemented

- Diverse Funding Sources: Securing support from government grants, private donations, and partnerships with healthcare organizations was crucial to maintaining a consistent resource flow for program maintenance and expansion.
- Efficient Resource Use: Prioritizing the development of tailored materials, training for staff, and maintaining technological platforms was essential. Allocating resources for regular program evaluation and feedback collection ensured continuous improvement.
- Community Partnerships: Collaborations with local healthcare providers, schools, and community organizations enhanced resource sharing and broadened the program’s reach. These partnerships facilitated referrals and increased program visibility.
- Scalability Planning: A modular approach allowed for replicating successful program elements in new locations. Pilot programs helped refine strategies before broader implementation, ensuring adaptability to different community needs and resources.

The program could achieve long-term sustainability and scalability with strategic funding and resource allocation, extending its benefits to a broader population.

## 5. Partners and Collaborations

KidsTUMove actively engages in collaborations across various sectors to enhance its impact. By collaborating with diverse organizations (e.g., parent initiatives, social foundations), institutions (e.g., regional schools, social pediatric centers), and professionals (e.g., primary care physicians, specialized children’s hospitals, health experts), KidsTUMove extends its reach, develops a multidisciplinary network and pools expertise and resources. These collaborative efforts promote a synergistic approach to advancing children’s health and well-being, fostering a culture of physical activity and education beyond the organization’s core initiatives. This strategic approach significantly amplifies KidsTUMove’s effectiveness in fulfilling its mission to promote physical activity and overall health among children. Through these partnerships, KidsTUMove continues to innovate and expand its outreach, ensuring that children receive the necessary support and opportunities to cultivate and sustain a healthy and active lifestyle. The program’s dedication to fostering collaborations underscores its holistic approach and steadfast dedication to making a lasting impact on children’s well-being. 

The KidsTUMove program has been selected through a Europe-wide call for proposals since early 2023 and is supported by the European Union. The ERASMUS-SPORT-2022 KidsTUMove goes Europe—cordially fit project in collaboration with four other countries, namely Italy, Spain, Portugal, and Greece, aims to raise awareness about the importance of integrating children and adolescents into sports activities.

KidsTUMove works closely with a parent’s association for children with CHD in South Tyrol, Italy. Through this partnership, KidsTUMove aims to provide specialized physical activity programs and support for children with heart conditions. 

KidsTUMove recognizes the importance of collaborating with hospitals and healthcare professionals to comprehensively support children’s health. Working alongside hospitals and pediatricians, KidsTUMove can ensure that children with specific health conditions or medical considerations receive appropriate guidance and support in their physical activity journey. This collaboration helps bridge the gap between healthcare and physical activity, promoting holistic well-being for children.

## 6. Discussion

This section discusses the principal findings of the holistic healthcare KidsTUMove project within the current literature and explores opportunities for its continuous development. Additionally, pathways to customize the KidsTUMove project as a model for other nations, and strategies to address challenges and facilitate implementation are examined. The project’s success is underscored by the ongoing expansion of services driven by demand from facilities serving children with chronic illnesses and affected families. Originating in clinics where children and adolescents with chronic diseases were initially offered exercise programs during hospital stays, the project has effectively evolved into a comprehensive holistic program to address the requirements of these individuals. This includes outpatient groups and decentralized summer and winter camps. Preliminary studies indicate the attainment of the WHO-recommended 60 minutes of daily physical activity, with participation in camps also showing potential to enhance quality of life [36,37,60,61]. 

### 6.1. Positive Impacts on Children

Specific sports activities and camps provide a unique environment where children with chronic diseases can engage in activities tailored to their specific needs, significantly improving their quality of life. Traditional camps often cannot accommodate these children due to the necessity for continuous medical supervision and specialized facilities [62]. KidsTUMove offers modular programs with structured, individualized activities in a safe space, strengthening self-awareness and socializing, and enhancing physical and emotional well-being. The natural environment, typical sports activities, age-appropriate groups, and the inclusion of siblings positively impact being a “normal child”. This range of goals is also mentioned in Békési et al. [63] and Kiernan et al. [64]: camps generally include “providing children with a fun-filled, age-appropriate experience where they can acquire activity-related skills; encouraging children to develop a self-sufficient attitude; enhancing self-esteem; providing opportunities for a sense of mastery and efficacy in peer relationships; and helping children learn about their illness either through formal education, or informal peer interaction”. 

Neville et al. highlight camps as “a place to call our own” and “interacting with and relate to individuals who are similar, while experiencing the joys typically associated with childhood” [65].

Additionally, Epstein et al. state, “It’s not just a camp” but “the healing and developmental power of play, finding acceptance and fit, grief as something to live with versus “get over,” storytelling as a means of reshaping and understanding traumatic experiences, and the solidarity of the community as one that creates intense, healing bonds” [66].

Creating a respectful environment in a safe space, KidsTUMove helps children achieve success and build confidence. The supportive and inclusive environment enables them to develop a positive self-image, which is often compromised in regular social settings, as mentioned in the studies of Békési et al. [63] and in an overview of 15 European countries investigated by Kiernan et al. [64].

By combining children with chronic diseases with their healthy friends and siblings, along with the support of an interdisciplinary team, KidsTUMove fosters the development of social contacts and peer groups. Isolation is a common issue among children with chronic diseases due to frequent medical appointments and physical limitations. Specialized camps create opportunities for these children to form meaningful social connections with peers who understand their experiences [67,68,69]. This fosters a sense of belonging and enhances social skills, which are crucial for overall development [70,71,72].

### 6.2. Benefits to Families

Specialized programs for children with chronic diseases benefit the children and have a profound impact on their families. For this reason, the involvement of parents is a crucial component of KidsTUMove. Parents gain confidence in managing their child’s condition and experience respite from the daily caregiving responsibilities. Additionally, siblings of affected children can connect with peers in similar situations, providing emotional support and understanding within the family unit [72]. Therefore, parent consultations and close collaboration are essential within the KidsTUMove project.

### 6.3. Comparative Analysis with Other Offers in Europe

Tackling chronic diseases is an essential topic in Europe [73]. In Germany, various initiatives exist to support children with chronic diseases, including hospital-based programs and outpatient rehabilitation services [74,75,76]. However, specialized modular programs like KidsTUMove provide a more holistic approach by integrating medical care with recreational activities in a natural setting with an interdisciplinary team of experts integrated into a university system. This combination provides a more comprehensive support system that addresses the children’s and families’ physical and emotional needs. Across Europe, the availability and quality of specialized camps for children with chronic diseases vary. However, they all emphasize the importance of offering tailored programs [72,77,78]. However, the extent and accessibility of these programs often depend on funding and regional healthcare policies, leading to disparities in service provision.

The need for 24-h medical supervision is a significant challenge in organizing specialized camps. Ensuring the availability of trained medical personnel and appropriate facilities is essential for the safety and well-being of the children. Collaborative efforts between healthcare providers and camp organizers can help address this issue by pooling resources and expertise [79,80]. 

Creating an environment that is both safe and conducive to the needs of children with chronic diseases requires careful planning and substantial resources. This includes accessible facilities, trained staff, and appropriate recreational activities [80]. Investment in training programs for staff and the development of standardized protocols can enhance the quality and safety of these camps. Therefore, as already mentioned, a trainer manual is being developed by experts from five European countries.

Securing financial support from government agencies, non-profit organizations, and private donors is vital to ensure the sustainability and accessibility of these programs. Innovative funding models, such as public–private partnerships and community fundraising initiatives, can provide the necessary financial backing. Therefore, public relations work is very important in our project to keep receiving new funding. The challenges of the project are presented below (Table 1). In addition, the solution strategies and the practical implementation are shown.

Given that the group of participants is very heterogeneous regarding chronic conditions with or without residual findings, age, and gender, no large cohort or comparison groups exist. Additionally, reliance on self-reported measures will pose a challenge in future studies to collect representative data. To generate specific statements about the impact of KidsTUMove camps and sports groups, it is aimed to recruit a larger number of participants for the program and to conduct a follow-up study of the participants for future research or program development. This is usually only possible if a large organization, such as a university or an association, is involved.

While numerous reviews highlight the positive short-term impacts of specialized camps, there is a need for more structured and long-term research. Each offer is unique in its design and implementation, making it challenging to generalize findings. Future research should focus on standardized outcome measures and longitudinal studies to better understand the long-term benefits and effectiveness of these programs.

## 7. Conclusions

KidsTUMove has demonstrated the feasibility of offering physical activity opportunities for children with chronic diseases. It is possible to provide care for children with chronic diseases within a safe and effective framework, albeit with some effort. Thus, no gap needs to persist for the target group. By facilitating engagement in physical activity, the program has contributed to enhancing the health, quality of life, and self-esteem of children affected by chronic diseases. The program’s success has spurred collaborations with other European countries, underscoring the necessity for European physical activity initiatives tailored to children with chronic diseases. Nonetheless, challenges remain in realizing comprehensive exercise programs for every child and adolescent with a chronic disease in Germany. These challenges encompass establishing quality standards and ensuring the financial sustainability of exercise programs. Future research endeavors should concentrate on standardized outcome measures and longitudinal studies to gain deeper insights into the long-term benefits and efficacy of these interventions.

Furthermore, given the growing number of forthcoming exercise programs, adequately trained coaches must be prepared to ensure safety and uphold quality standards. To address this need, a trainer manual for specialized qualification courses, specifically tailored for children and adolescents with chronic diseases, is presently under development. In summary, future efforts for children and adolescents with chronic diseases entail establishing quality standards, fostering multicenter collaborations, and providing specialized staff training to ensure safety and uphold quality standards in exercise programs.

## Figures and Tables

**Table 1:** Challenges and possible solution strategies within the KidsTUMove project.

Modular Program	Topic	Identified Challenges and Constraints	Possible Strategies
Regular sports activities: Move it—integrative sports group, KidsTUMove climbing group	Location	Sports hall availability	Integration into existing sports clubs
	Personnel	Qualified staff	Recruitment of trainers through associations, education facilities, and universities (e.g., integration into teaching)
	Financial Resources	Costs for material and qualified trainers	Raising donations in relation to specific items; course fees or membership fees; health insurance support
	Acquisition	Limited attention from the public/community	Increase visibility through flyers, website, personal network; dissemination strategy development; participation in local events, conferences and congresses
	Medical Care	Medical emergencies, participant-related selection criteria: >3-6 months post-operation, sports clearance certificate, no psychological impairments, independent daily living (no caregiving activities assumed)	Pediatrician at venue; first aid kit and defibrillator available; close collaboration with parents
	Insurance	Vulnerable participants (higher sum insured), accidents	Insurance through the association with special medical conditions
	Data Protection	High data security and duty of confidentiality	Data encryption and personal access guidelines
	Schedule	Conflict with school and other leisure activities	Consideration of school commitments, consultation with parents, adaptable schedule
	Evaluation	Effectiveness of the program	Status quo test and longitudinal analysis in the course of training (quality of life, motor skills, physical activity)
Camps and Events: KidsTUMove summer and winter camps, KidsTUMove weekend events	Location	Suitable accommodation and sports facilities with opportunities for indoor and outdoor sports courses. Easily accessible medical care near the venue	Cooperation with foundations and clubs that provide their facilities
	Personnel	Qualified staff	Recruitment of trainers through associations, universities (e.g., integration into teaching); third-party projects; volunteers
	Financial Resources	Integration of long-term sponsors, regional supporters	Long-term cooperation with foundations, associations and parents’ initiatives; participant fees
	Medical Care	Availability of pediatrician. Continuous and monitored administration of medication. Participant-related special selection criteria: >3-6 months post-operation, sports clearance certificate, no psychological impairments, independent daily living (no caregiving activities assumed)	24-h doctor available at venue; medication plan and administration by medical staff on-site; clinics/emergency services informed near venue; contact with medical care centers and treating physicians; emergency equipment (e.g., defibrillator) on-site
	Insurance	Vulnerable participants (higher sum insured), accidents	External insurance company for participant insurance
	Acquisition	Limited attention from the public/community	Increase visibility through flyers, website, personal network; dissemination strategy development; participation in local events, conferences and congresses
	Data Protection	High data security and duty of confidentiality	Data encryption and personal access guidelines
	Evaluation	Effectiveness of the program, Quality analysis	Pre/post tests (quality of life, satisfaction, motor skills, physical activity), supervision
Online activities: KidsTUMove goes online!	Location	Adequate space with good lighting, available capacity, and sufficient electrical outlets	Cooperation with universities or foundations
	Personnel	Qualified staff	Recruitment of trainers, students, and trainees through associations or university (e.g., through integration into teaching); third-party projects; volunteers
	Financial Resources	Costs for internet platforms, media equipment and technical expertise of employees	Equipment on a donation basis; equipment rental; cooperation with foundations
	Medical Care	Supervision difficult: no personal contact	Safety guidelines for supervised online training
	Schedule	Timing, compatible with school schedules, pandemic issues	Hybrid offer; live sessions; dispatching material packages for the offerings
	Acquisition	Expansion of target group, accessibility of participants	Homepage setup; using network
	Data Protection	High data security and duty of confidentiality, internet security, and traceability on the web	Data encryption and personal access guidelines
	Evaluation	Effectiveness of the program, Quality analysis	Progress controls via online forms (quality of life, satisfaction, physical activity)

## Data Availability

No new data were created or analyzed in this report. Data sharing is not applicable to this report.
